# Supplementary material for: Barriers and Facilitators to Proactive Deprescribing in Saudi Hospitals: A Qualitative Study Using the Theoretical Domains Framework
Source: Healthcare (Basel). 2025 May 28;13(11):1274. doi: 10.3390/healthcare13111274 (PMC12155129; doi:10.3390/healthcare13111274)
Supplement: Supplementary file 1 [file healthcare-13-01274-s001.zip › healthcare-3596442-supplementary.pdf]

healthcare

MDPI

Table S1. COREQ Checklist: Consolidated Criteria for Reporting Qualitative Research (32-Item Checklist).

Study Title: Barriers and Facilitators to Proactive Deprescribing Among Physicians in Saudi Hospitals: A Qualitative Study Using the Theoretical Domains Framework

Item	Item number	Response	Page no.
Domain 1: Research team and reflexivity			
Interviewer/facilitator	1	The lead researcher conducted the interviews.	
Credentials	2	PhD in Pharmacy Practice.	
Occupation	3	Assistant Professor in Pharmacy Practice at the time of the study.	
Gender	4	Male.	
Experience and training	5	Trained in qualitative interviewing and experienced in pharmacy practice research.	
Relationship established	6	No prior relationship was established before the interviews.	
Participant knowledge of the interviewer	7	Participants were informed that the interviewer was a researcher studying deprescribing practices.	
Interviewer characteristics	8	The interviewer maintained neutrality, used reflexive journaling, and applied consistent semi-structured techniques.	
Domain 2: Study design			
Methodological orientation and theory	9	Theoretical Domains Framework (TDF).	
Sampling	10	Purposive sampling of physicians experienced with polypharmacy management.	
Method of approach	11	Contacted via phone and email.	
Sample size	12	27 participants.	
Non-participation	13	No specific non-participation data reported.	
Setting of data collection	14	Interviews were conducted remotely or in-person based on convenience.	
Presence of non-participants	15	Only the participant and researcher were present during interviews.	
Description of sample	16	Diverse in age, gender, specialty, experience, and region.	
Interview guide	17	Developed from literature and expert consultation, and pilot-tested with academic peers.	
Repeat interviews	18	No repeat interviews were conducted.	
Audio/visual recording	19	All interviews were audio-recorded.	
Field notes	20	Field notes and memos were maintained.	
Duration	21	Interviews lasted 30–60 minutes.	
Data saturation	22	Achieved by interview 27, as no new themes emerged.	
23. Transcripts returned	23	Transcripts were not returned to participants; peer debriefing ensured the credibility of the findings.	
Domain 3: Analysis and findings			
24. Number of data coders	24	One coder (the lead researcher) conducted analysis.	
25. Description of the coding tree	25	Themes derived based on the 14 domains of the TDF.	
26. Derivation of themes	26	Themes were deductively derived using the TDF framework.	

27. Software	27	Data were manually coded without software.
28. Participant checking	28	Participants did not review the findings.
29. Quotations presented	29	Verbatim participant quotes were used and attributed by ID.
30. Data and findings consistent	30	Clear alignment between quotations and reported themes.
31. Clarity of major themes	31	Major themes structured and discussed in Results and Discussion.
32. Clarity of minor themes	32	Minor and divergent views included in supplementary file discussion.

Table S2: Illustrative Variation in Perspectives Across Medical Specialities.

While the primary analysis was organised around behavioural domains using the Theoretical Domains Framework (TDF), a secondary review of transcripts revealed informal variations in deprescribing perspectives across clinical specialities. This table summarises illustrative differences observed during thematic analysis. These findings were not derived from formal subgroup comparison but offer exploratory insight for future research.

Specialty	Notable Emphasis or Concern
Neurology	Heightened concern about withdrawal effects, especially in patients with epilepsy or neurological instability.
Paediatrics	Strong caregiver resistance; emphasis on maintaining long-term medication regimens to reassure families.
Internal Medicine	Focus on polypharmacy risks and multimorbidity, with an interest in clear deprescribing protocols.
Rehabilitation Medicine	Emphasis on reducing sedative use to support mobility and improve cognitive recovery.
Oncology	Cautious approach to deprescribing in palliative care; medico-legal concerns and emotional implications noted.

Table S3. Interview Guide Based on the Theoretical Domains Framework (TDF).

This semi-structured interview guide was developed using the 14-domain Theoretical Domains Framework (TDF), informed by literature review and expert consultation. The purpose was to explore a comprehensive range of behavioural determinants influencing physicians' engagement with proactive deprescribing in hospital settings. While all 14 TDF domains were considered during guide development and interview questioning, the final analysis identified seven key domains that were most strongly represented in the transcripts. Sample questions below illustrate how selected domains were explored during the interviews:

TDF Domain	Sample Interview Questions
Environmental Context and Resources	What institutional policies or tools support or hinder your ability to deprescribe? Do you feel you have enough time during consultations to review and discontinue medications when necessary? How do electronic health records or pharmacist support affect your deprescribing decisions?
Social Influences	How do colleagues, pharmacists, or geriatricians influence your decisions regarding deprescribing? Have you encountered support or resistance from patients or caregivers when trying to discontinue medications?
Beliefs About Capabilities	How confident do you feel in deciding to deprescribe medications for complex patients? Have you received any training that helped build your confidence in deprescribing?
Social/Professional Role and Identity	Do you consider deprescribing to be part of your responsibility as a physician? How does your role in the hospital influence your decisions around medication discontinuation?
Skills	What skills are crucial for successfully deprescribing medications? How have your communication skills helped (or not) in discussing deprescribing with patients or caregivers?
Beliefs About Consequences	What do you believe are the benefits or risks of deprescribing? Have you experienced any adverse outcomes or successes after deprescribing a medication? How did that influence your practice?
Knowledge	What do you know about deprescribing practices or guidelines? Where did you learn about deprescribing? Are there any tools or resources you rely on?
